# Chitosan–Hydroxyapatite Composite Layers Generated in Radio Frequency Magnetron Sputtering Discharge: From Plasma to Structural and Morphological Analysis of Layers

**DOI:** 10.3390/polym12123065

**Published:** 2020-12-21

**Authors:** Dragana Biliana Dreghici, Bogdan Butoi, Daniela Predoi, Simona Liliana Iconaru, Ovidiu Stoican, Andreea Groza

**Affiliations:** 1 National Institute for Laser, Plasma and Radiation Physics, 409 Atomistilor Street, P.O. Box MG36, Magurele, 077125 Bucharest, Romania; dragana.dreghici@inflpr.ro (D.B.D.); bogdan.butoi@inflpr.ro (B.B.); ovidiu.stoican@inflpr.ro (O.S.); 2 National Institute of Materials Physics, Atomistilor Street, No. 405A, P.O. Box MG 07, Magurele, 077125 Bucharest, Romania; dpredoi@gmail.com (D.P.); simonaiconaru@gmail.com (S.L.I.)

**Keywords:** chitosan–hydroxyapatite composite coatings, radio frequency magnetron sputtering plasma discharge, grain-like structure surfaces

## Abstract

Chitosan–hydroxyapatite composite layers were deposited on Si substrates in radio frequency magnetron sputtering discharges. The plasma parameters calculated from the current–voltage radio frequency-compensated Langmuir probe characteristics indicate a huge difference between the electron temperature in the plasma and at the sample holder. These findings aid in the understanding of the coagulation pattern of hydroxyapatite–chitosan macromolecules on the substrate surface. An increase in the sizes of the spherical-shape grain-like structures formed on the coating surface with the plasma electron number density was observed. The link between the chemical composition of the chitosan–hydroxyapatite composite film and the species sputtered from the target or produced by excitation/ionization mechanisms in the plasma was determined on the basis of X-ray photoelectron spectroscopy, Fourier transform infrared spectroscopy and residual gas mass spectrometry analysis.

## 1. Introduction

Coatings based on hydroxyapatite are used in different biomedical applications such as bone substitution and bone regeneration due to the similarities between their chemical composition and the one corresponding to the inorganic component of bone structures [1,2,3]. Hydroxyapatite (HAp), Ca_10_(PO_4_)_6_(OH)_2_), is a calcium phosphate biomaterial with high biocompatibility, bioactivity and osteoconductivity that can be found in human bones [1,2,3], being extensively used for bone tissue engineering. Thin films of hydroxyapatite that cover implants and scaffolds for tissue substitution promote cells and proteins adhesion, are used to simulate osteoblast activity and increase the mechanical strength of the substrates.

Previous publications reported improved mechanical, physicochemical and biological properties of hydroxyapatite by synthesis with chitosan [4,5]. Chitosan ((C_6_H_11_NO_4_)_n_) is a polymer with bioactive, bioabsorbable and antimicrobial properties, being nontoxic to the human organism. It is used in biomedical applications for scar-free wound healings or anti-inflammation therapy due to its capacity to slowly release medicines [5].

Chitosan–hydroxyapatite composite coatings have been obtained, until now, by: immersion [6], micro-arc oxidation [7], sol gel [8], biomimetic or electrophoretic deposition methods [9]. However, magnetron sputtering, thermal plasma spraying, pulsed laser deposition or electrospinning methods are extensively used for calcium phosphate-based coating depositions on polymeric or metallic substrates [1,2,3].

In our previous works, we showed that at low radio frequency (rf) working powers, in magnetron sputtering plasma discharges, bioceramic multicomponent coatings for biomedical applications can be synthetized [10,11,12]. In [10], we showed that in rf magnetron discharge at about 1.2 × 10^−2^ mbarr and 50 W rf power, after 4 h deposition time, regular granular-like structures on the surface of calcium phosphate layers deposited on Ti substrates can be generated. These structures were not observed anymore at the pressure of 4.6 × 10^−3^ mbarr, with the layers presenting smooth surfaces. Previously [1,10], many authors reported on the grain-like structure of the surfaces of hydroxyapatite coatings generated in magnetron sputtering discharges when the substrate holder is grounded [3]. In fact, these surface morphologies reflect the columnar grains growth perpendicular to the substrate structure of the hydroxyapatite coatings.

RF magnetron sputtering of polymers is of high interest for technological applications [13] because plasma-polymerized micron- to nano-sized particles can be generated as a function of experimental conditions. On substrates, plasma polymer nanoparticles can agglomerate and lead to the growth of nanostructures and nonporous films. Plasma polymers are also attractive as their architectural structures are highly cross-linked with branches impossible to generate by chemical methods [13,14].

This work reports on the deposition of chitosan–hydroxyapatite composite layers (HApCs) in radio frequency magnetron sputtering discharge. Information of plasma parameters including the electron energy distribution function contributes to understanding the mechanism behind the surface topography and molecular structure of the layers. The influence of the polymer molecules sputtered from the target and consequently vaporized into the plasma on the fragmentation of hydroxyapatite was established by residual gas mass spectrometry.

The chemical composition and bindings between hydroxyapatite and chitosan were investigated by X-ray photoelectron spectroscopy (XPS) and Fourier transform infrared spectroscopy (FTIR). More than that, the comparison between the infrared (IR) spectra of the sputtering target and the IR spectra of the films suggests that the processes that are encountered in the plasma during the deposition processes influence the molecular structure of the film. The scanning electron microscopy (SEM) surface analysis revealed the morphological features of the coatings and the particle size distribution of their grain-like structure. The elemental composition of the chitosan–hydroxyapatite composite films was also identified by energy dispersive X-ray spectroscopy (EDS).

## 2. Materials and Methods

### 2.1. Materials

Surface modification of nano-hydroxyapatite with chitosan was obtained using (NH_4_)_2_HPO_4_ (ammonium hydrogen phosphate from Sigma Aldrich, St. Louis, MO, USA, ≥99.0%), Ca(NO_3_)_2_∙4H_2_O (calcium nitrate tetrahydrate-Sigma Aldrich, St. Louis, MO, USA, ≥99.0%), NH_4_OH (ammonium hydroxide, Sigma Aldrich, St. Louis, MO, USA, 25% NH_3_ in H_2_O), chitosan (Sigma Aldrich, St. Louis, MO, USA), C_2_H_5_OH (ethanol absolute from Merck, New Jersey, USA, ≥99.5%) and de-ionized water.

### 2.2. Synthesis of Chitosan–Hydroxyapatite Composites

To obtain the chitosan–hydroxyapatite composites, the solution (NH_4_)_2_HPO_4_ was added to the mixture of an aqueous solution of Ca(NO_3_)_2_·4H_2_O and chitosan in the ratio 3:2. During the synthesis, the pH was maintained at 10 with ammonium hydroxide solution. The stoichiometric ratio of Ca to P was 1.67. The obtained precipitate was filtered and washed four times to bring the pH to 7. The resulting precipitate obtained was dried at 150 °C to get the HApCs composite [15]. The HApCs powder obtained from the dry product at 150 °C was used to manufacture the target. The target of 2 inches in diameter was obtained by mechanical pressing of the HApCs composite nano-powder for a few minutes and dried at 150 °C.

### 2.3. Synthesis of Chitosan–Hydroxyapatite Layers

HAp–chitosan composite layers were deposited on Si substrates in radio frequency (rf) magnetron sputtering discharge in Argon gas. The experimental set-up was described in detail in [10]. The experimental working parameters were the following: 30 W rf power; Ar gas working pressures between 2 × 10^−3^ and 1.2 × 10^−2^ mbarr (base pressure ~ 10^−5^ mbarr); Ar gas flows in 0.8–4.5 sl_n_/min range; 4 cm magnetron source–substrate holder distance; and 20 h deposition time. The deposition rate varied between 0.03 and 0.05 Å/s as a function of the Ar gas working pressure which indicates a layer thickness of 216–360 nm. The substrate holder was grounded without being cooled or heated. The temperature at the substrate holder did not exceed 250 °C (523 K) during the deposition process and was measured using a single-ended thermocouple probe (acquired from K.J.Lesker Company) [10]. Si substrates (10 × 10 × 1 mm) with mirror-like surfaces were positioned centrally on the holder support.

### 2.4. Characterization Methods

The electron distribution function, electron temperature and electron number density were determined from the current–voltage (IV) characteristics measured by using a cylindrical homemade rf-compensated Langmuir probe inserted in the middle of the plasma, at a distance of 2 cm from the magnetron source, at a point where the intensity of the magnetic field is approximately zero. It consists of a tungsten wire with a 0.1 mm diameter and a length of 10 mm. The IV characteristics were acquired by applying ± 100 V on the Langmuir probe. The rf-compensated Langmuir probe was designed in accordance with [16]. The probe tip was immersed into the plasma for a short period of time before starting the deposition process, only for the IV characteristic measurements. The probe tip was cleaned after each measurement and several sets of IV curves were recorded for data accuracy. A mean value of the electron temperature T_e_ was calculated as T_e_ (eV) = 1/slope of the plot of natural logarithm of the electron current intensity versus the probe potential [17].

The Druyvesteyn electron energy distribution function of the plasma and the electron number density were calculated using the following formulas [17]:

(1) $$f\left(\varepsilon \right)=\;\frac{2}{e^{2}A}{\left(\frac{2m\varepsilon }{e}\right)}^{1/2}\frac{d^{2}I}{dV^{2}}$$

(2) $$n_{e}=\int_{0}^{\infty }f\left(\varepsilon \right)d\varepsilon$$

where *ε* is the electron energy, *e* is the electron charge, *A* is the area of the cylindrical probe, *m* is the electron mass and *d^2^I/dV^2^* is derived from the IV Langmuir probe characteristic.

By using a QMS200 spectrometer (Pheiffer Vacuum Company, Aßlar, Germany) coupled with the deposition vacuum chamber [10], the plasma residual gas composition was identified. The mass spectra were recorded during the deposition process of the HApCs layers. The pressure inside the vacuum chamber of the mass spectrometer during the analysis was maintained at 10^−5^–10^−6^ mbarr by means of a mechanically controlled valve.

A Perkin Elmer SP-100 spectrometer (Waltham, MS, USA) working in the 4000–400 cm^−1^ spectral range and provided with an attenuated total reflection unit was used for investigation of the molecular structure of the HApCs layers. The interconnections between the chemical bonds characteristic both to HAp and chitosan were identified after we performed the curve fitting analysis of the HApCs IR spectrum in the spectral range specific to P-O bonds, respectively, 1200–800 cm^−1^ and 650–500 cm^−1^. The curve fitting procedure was presented in detail in [18].

XPS analyses were performed using a K-Alpha Thermo Scientific (ESCALAB™ XI+, East Grinstead, UK) spectrometer equipped with a 180° double focusing hemispherical analyzer. The C 1s peak at 284.6 eV was used for peak positions calibration. The full spectra of the films were recorded with a pass energy of 50 eV. High-resolution spectra of XPS lines were measured with 20 eV pass energy and 0.1 eV energy step size for the evaluation of elemental bonding states of the HApCs layers. The spectra acquisition and data processing were performed by using advanced Advantage data software.

The simultaneous deposition of chitosan and hydroxyapatite in magnetron sputtering discharge leads to depositions of coatings with grain-like structures. These typical features of the surface of the HApCs coatings were investigated by scanning electron microscopy (SEM) using an FEI Inspect S scanning electron microscope (Hillsboro, Oregon, OR, USA) in both high- and low-vacuum modes. The microscope has attached inside an EDAX Inc. SiLi detector for elemental compositional analysis using energy dispersive X-ray spectroscopy (EDS).

## 3. Results

### 3.1. Plasma Characterization by Langmuir Probe Electrical Measurements

The plasma parameters extracted from the rf-compensated IV Langmuir probe characteristics measured in various experimental conditions are presented in Table 1. The data indicate the dependence of the electron temperature and electron number density on the Ar gas working pressure and applied rf power. It can be observed that the T_e_ decreases and n_e_ increases as the Ar gas working pressure increases. These results are in good agreement with previous studies [16].

**Figure 1:**
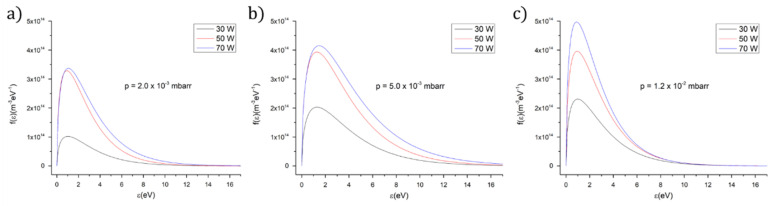
Dependence of electron energy distribution function on applied radio frequency (rf) power for: (**a**) *p* = 2 × 10^−3^ mbarr; (**b**) *p* = 5 × 10^−3^ mbarr; (**c**) *p* = 1.2 × 10^−2^ mbarr.

The Druyvesteyn electron energy distribution functions were calculated using the experimental measured current–voltage rf-compensated Langmuir probe characteristics and Formula (1). The graphs from Figure 1 indicate no major differences between the evolution of the electron energy distribution function with the rf power for different Ar gas working pressures.

### 3.2. Mass Spectra Analysis of Synthesis Plasma

The neutral components of the deposition plasma generated in the rf magnetron discharge are mainly due to the sputtering from the solid target of the organic and inorganic species characteristic of chitosan and HAp chemical structures. They have been identified by mass spectra analysis of the residual gas extracted from the vacuum chamber. A comparison between the mass spectra acquired during the deposition process of the HApCs films and after the plasma ceasing was also performed.

Previously, in [10], the molecular fragments characteristic of the residual gas extracted from a calcium phosphate plasma were presented. Likewise, CaO^+^, Ca^2+^, PO and POH neutral and positive ion species were found in the calcium phosphate magnetron sputtering plasma discharge by optical emission spectroscopy [19].

**Figure 2:**
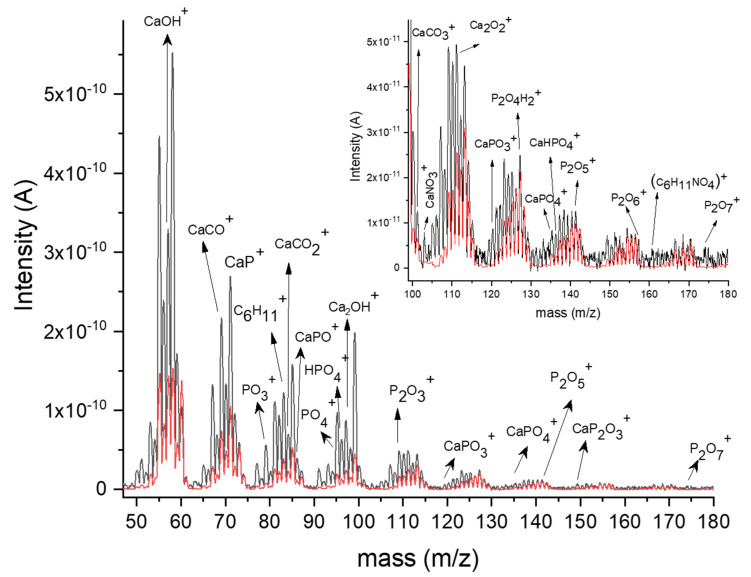
Mass spectrum of the residual gas extracted from the vacuum chamber during plasma on (black line) and plasma off (red line).

Figure 2 presents the mass spectra of the residual gas extracted from the deposition chamber during and after the ceasing of the plasma. The dissociation of the chitosan organic molecules into the plasma and their association with Ca and P atoms give birth to a lot of byproducts that slow down an accurate identification of the HAp species involved in the deposition process. Moreover, the breakage of certain bonds characteristic of HAp (Ca_10_(PO_4_)_6_(OH)_2_) and chitosan (C_6_H_11_NO_4_)_n_ chemical structures is possible in plasma as the electron energy distribution function (see Figure 1) indicates: Ca-Ca (0.15 eV), Ca-O (4.8 eV), P-O (6 eV), C-H (3.5 eV), O-H ( 4.4 eV), N-O (6.5 eV), N-H (3.2 eV), C-N (7.9 eV), C-O (11 eV) and C=O (7.7 eV) [19]. Usually, the dissociation energies of organic molecular fragments do not exceed the dissociation energies of chemical bonds between two atoms [20].

The organic molecular fragments detected with a residual gas mass spectrum analyzer are divided in two categories: fragments produced in the plasma and fragments generated due to electron ionization inside the spectrometer. The differences between these two types of molecular species can be highlighted considering that C_n_H_2n-1_^+^ and C_n_H_2n+1_^+^ ion types appear mainly due to ionizations inside the mass spectrometer [21], and only the C_n_H_2n_^+^ ions come from the plasma. For the proper identification of molecular fragments, the association processes between the organic molecules characteristic of chitosan and those specific to hydroxyapatite during the plasma deposition were also considered.

The following molecular fragments were identified (see Figure 2): Ca^+^ (40 *m/z*), Ca_2_^+^ (80,15 *m/z*), Ca_3_^+^ (120,23 *m/z*), CaOH^+^ (57.078 *m/z*), Ca_2_OH^+^ (97,15 *m/z*), Ca_3_OH^+^ (137.23 *m/z*), Ca_2_O^+^ (96.05 *m/z*), CaCO_3_^+^ (100 *m/z*), CaNO_3_^+^ (110.77 *m/z*), Ca_2_O_2_^+^ (111.95 *m/z*), PO_3_^+^ (78.94 *m/z*), PO_4_^+^ (94,93 *m/z*), P_2_O_3_^+^ (109.64 *m/z*), P_2_O_4_^+^ (125,54 *m/z*), P_2_O_5_^+^ (141.44 *m/z*) and P_2_O_7_+ (173.2 *m/z*). Typical calcium phosphate fragments: CaP^+^ (71,048 *m/z*), CaPO^+^ (86.9 *m/z*), CaPO_3_^+^ (118,8 *m/z*), CaPO_4_^+^ (134,73 *m/z*), CaP_2_O_3_^+^ (149,9 *m/z*) and Ca_3_PO^+^ (167,19 *m/z*), were also detected. Such positive molecular ions specific to calcium phosphate were also identified by time-of-flight secondary ion mass spectrometry analysis of some stones based on calcium phosphate compounds [22,23,24,25,26,27].

Beside these fragments belonging to hydroxyapatite, we assigned positive ions characteristic of the chitosan chemical structure (C_6_H_11_NO_4_)_n_: C_6_H_11_^+^ (83 m/z), (C_6_H_11_NO_4_)^+^ (160,96 m/z) and NO_4_^+^ (77.96 m/z).

In addition to the decrease in ion peak intensities, the recordings of both mass spectra when the plasma is on (black line) and in the first two minutes after the stopping of the rf magnetron discharge (red line) do not reveal major differences between the molecular species sputtered from the target (and involved in the deposition process) and those generated by dissociation/association reactions in the plasma (see Figure 2).

The findings of the plasma species during the deposition process are an essential step in the identification of HAp and chitosan molecules sputtered from the target, plasma byproducts and the molecular structure of the coatings.

### 3.3. XPS Analysis of Hydroxyapatite–Chitosan Coatings

**Figure 3:**
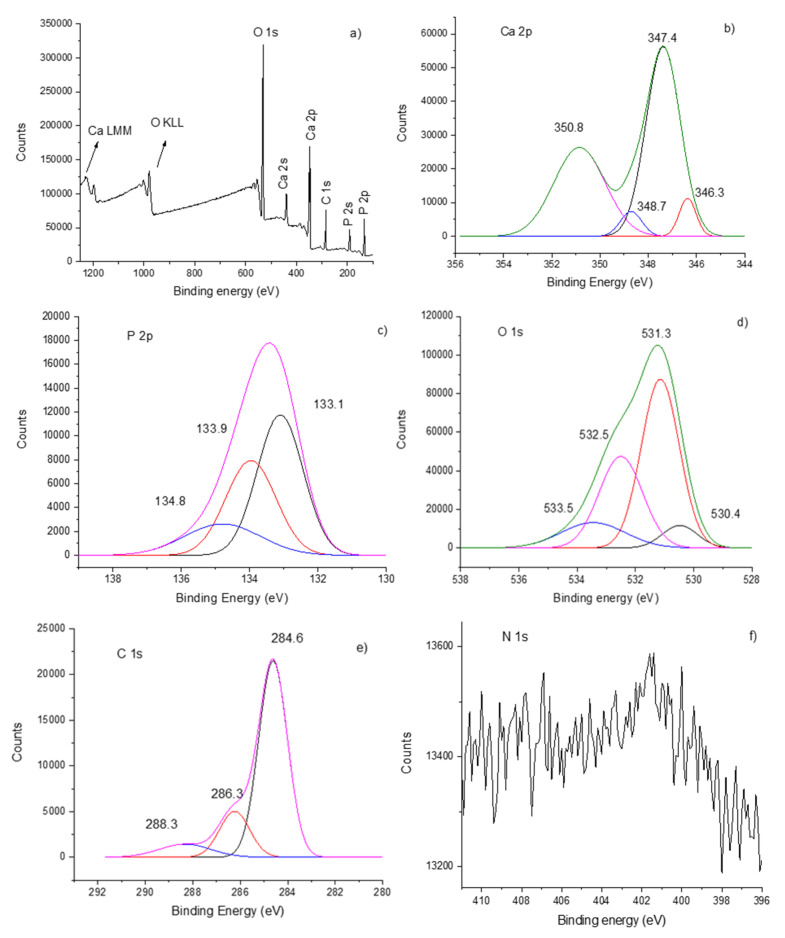
XPS data of HApCs coatings deposited on Si substrates at 5 × 10^−3^ mbarr Ar gas working pressure: (**a**) survey spectrum; (**b**) Ca 2p; (**c**) P 2p; (**d**) O 1s; (**e**) C1s; and (**f**) N 1s high-resolution lines.

The surface chemical state of the films generated in the rf magnetron sputtering discharge as well as the interconnected chemical bonds between chitosan and hydroxyapatite were investigated by X-ray photoelectron spectroscopy (XPS). The recorded XPS full spectrum indicates the presence of Ca, P, O, N and C elements (see Figure 3) and the high-resolution Ca 2p, P 2p, O 1s, N 1s and C 1s XPS lines give valuable information about the chemical structure of the coatings.

The Ca 2p high-resolution XPS spectrum (see Figure 3b) shows the doublet band which is characteristic of calcium-oxygen compounds [17,28]. The Ca 2p_3/2_ XPS line centered at 347.4 eV binding energy is attributed to the following Ca-O bindings in hydroxyapatite: Ca-O, Ca-OH and Ca-Ca [17]. The subpeak located at 346.3 eV indicates the Ca-CO_3_ bonds. The Ca 2p_1/2_ line appears at 350.8 eV and the deconvoluted peak from 348.7 eV indicates the formation of Ca-NO_3_ bonds [28]. The Ca-CO_3_^+^ and Ca-NO_3_^+^ ions were also detected in the mass spectrum of the residual gas extracted from the vacuum chamber during the plasma deposition process (see Figure 2).

Figure 3c presents the deconvolution spectrum of the P 2p XPS line. The P 2p_3/2_ line positioned at 133.1 eV indicates the bonding of phosphor to oxygen in the (PO_4_)^3−^ group in the hydroxyapatite structure and the P 2p_1/2_ XPS line is revealed after 0.8 eV, namely, at 133.9 eV. The 134.8 eV deconvoluted XPS peak indicates the formation, during the deposition process, of P-O bindings in P_2_O_5_ [17]. This compound was also identified in the mass spectrum from Figure 2.

The O 1s XPS spectrum of the HApCs layers (see Figure 3d) is deconvoluted in four subpeaks characterizing the O chemical state. The peak located at 531.3 eV indicates the O-P binding in the (PO_4_)^3-^ group and the one from 530.4 eV can be attributed to O-C binding in the hydroxyapatite structure. The peaks located at 532.5 and 533.5 eV can be assigned to oxygen from the polysaccharide backbone of chitosan [29] and to hydroxide groups [28], respectively.

The C 1s peak contains three subpeaks (see Figure 3e) positioned at 284.6 eV, 286.3 eV and 288.3 eV. The first peak is typical of carbon bound to carbon or hydrogen [C-(C, H)] and the second peak is specific to carbon bound to oxygen or nitrogen [C-(O, N)] in chitosan [29] and hydroxyapatite structures. The 288.3 eV peak indicates carbon bound to oxygen in the CO_3_ group.

The N1s XPS high-resolution spectrum presented in Figure 3f indicates a band located at 401.3 eV. The intensity of the N1s XPS line is small as the nitrogen is the least in the structure of chitosan (C_6_H_11_NO_4_)_n_. Moreover, the low intensities of the 286.3 eV C1s XPS deconvoluted line (attributed to carbon bound to oxygen or nitrogen [C-(O, N)] in chitosan) and of the 348.7 eV deconvoluted XPS line (attributed to Ca-NO_3_) indicate that the retention of N-containing bonds at the coating surface is reduced. Some previous works [30] showed that the incorporation of nitrogen groups in coatings generated by the plasma technique is slower than in the case of oxygen and carbon groups. Further, it was proved [1,3,16] that the bombardment of growing a film with species ejected from the target (especially with negative oxygen ions) or produced in the plasma during the deposition process can cause the resputtering of certain elements.

For the recognition of all the chemical products formed in the layers, beside hydroxyapatite and chitosan, the mass spectra of the residual gas extracted from the deposition chamber (see Figure 2) were essential.

Similar XPS spectra were obtained for HApCs coatings generated at 2 × 10^−3^ mbarr and 1.2 × 10^−2^ mbarr Ar gas working pressures and 30 W rf power.

### 3.4. Fourier Transform Infrared Spectroscopy Analyses of Chitosan–Hydroxyapatite Composite Coatings

The IR molecular bands of the HAp structure belonging to vibrational modes of P-O bonds in [PO_4_]^3−^ groups are usually found at: 1100–1000 cm^−1^ (ν_3_), 960 cm^−1^ (ν_1_), 630–500 cm^−1^ (ν_4_) and 470 cm^−1^ (ν_2_) [10,31]. The IR bands in the 1100–1000 cm^−1^ and 630–500 cm^−1^ ranges are the most affected by the interaction/interlinking of the HAp atoms with different doping elements. Therefore, the curve fitting analysis of these IR bands is helpful in revealing the molecular structure of the newly formed composite compounds.

The characteristic molecular bands of chitosan are found in the 3500–2500 cm^−1^ and 1800–500 cm^−1^ spectral regions [5].

**Figure 4:**
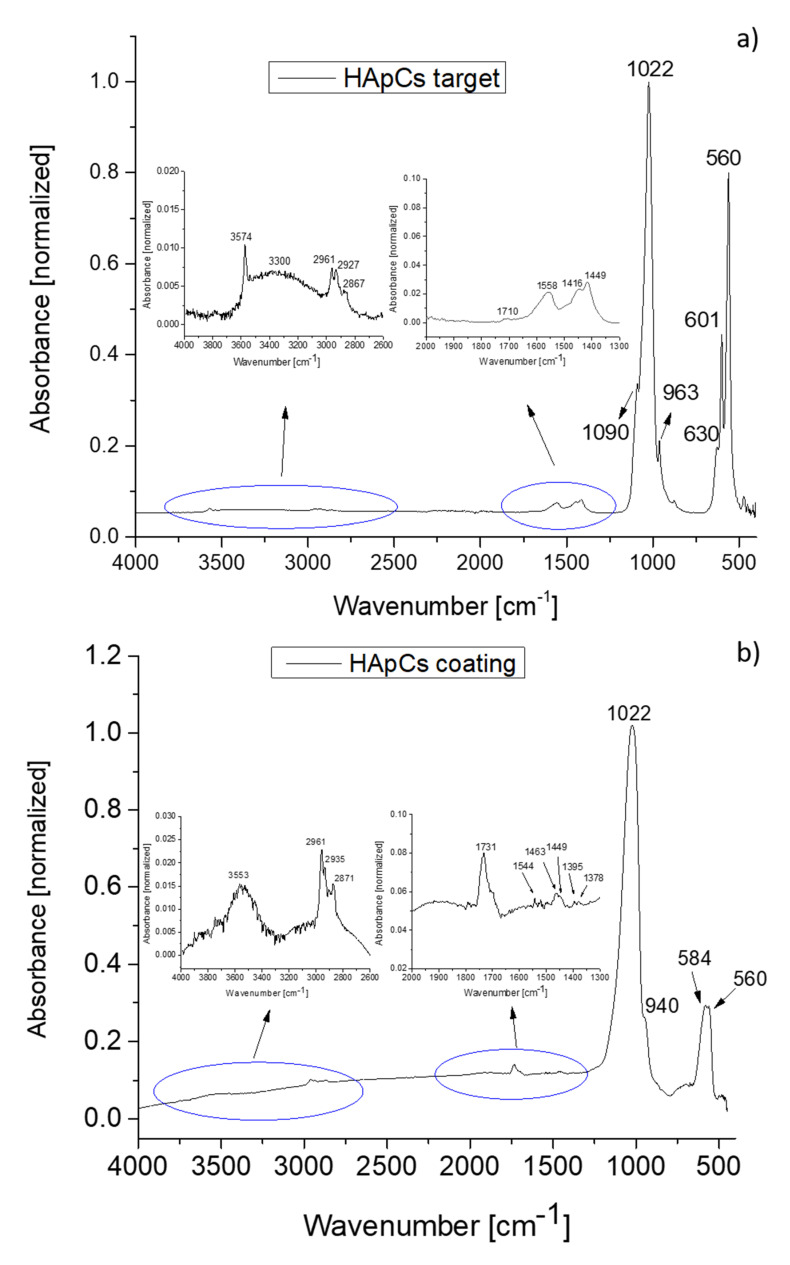

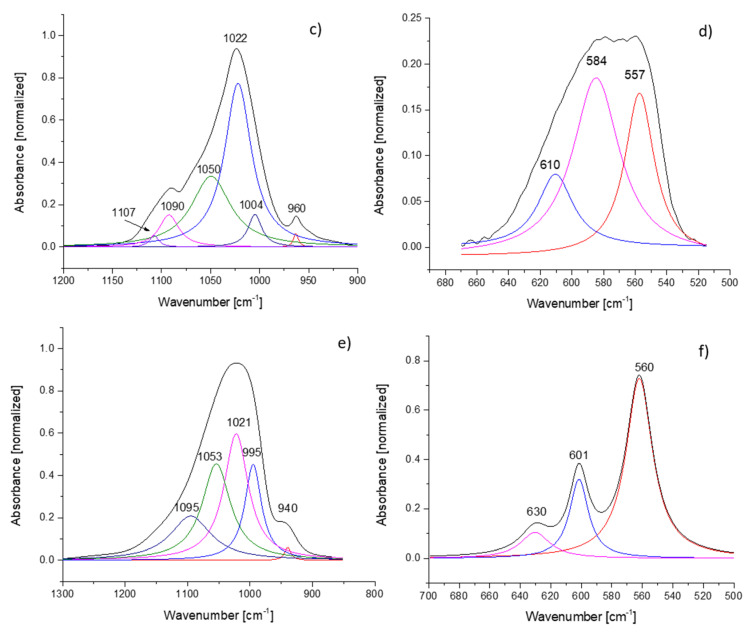
FTIR spectra of: (**a**) HApCs target; (**b**) HApCs coating deposited on Si substrate at 5 × 10^−3^ mbarr Ar gas working pressure; FTIR deconvoluted spectra of HApCs target and of HApCs coating deposited on Si substrate at 5 × 10^−3^ mbarr Ar gas working pressure in: (**c**,**e**) 1200–850 cm^−1^ range; (**d**,**f**) 680–500 cm^−1^ range.

The IR spectra of HApCs coatings (Figure 4b) generated by the rf magnetron sputtering technique were analyzed in comparison with the IR spectrum characteristic of the HApCs sputtering target (Figure 4a). Thus, it was established that the IR bands assigned to chitosan appear both in the IR spectra of the sputtering target and of the plasma generated films.

In the 4000–2600 cm^−1^ domain, there are shifts of the IR bands which correspond to amine and hydroxyl groups (see Figure 4b), as an indication of the interaction between the NH_3_ groups of chitosan with the OH groups of HAp during the plasma deposition process. The characteristic IR bands of N-H vibrations from 3574 cm^−1^ and O-H vibrations from 3300 cm^−1^ (Figure 4a) overlap and appear as one broad band centered at 3553 cm^−1^ in the spectrum of the film (Figure 4b). The IR bands at 2927 cm^−1^ (C-H asymmetric stretching in CH_2_) and 2867 cm^−1^ (C-H symmetric stretching in CH_3_) in the IR spectrum of the target are shifted to 2935 and 2871 cm^−1^ in the spectrum of the film, while the position of the IR band from 2961 cm^−1^ (C-H asymmetric stretching in CH_3_) remains unchanged.

In the 1800–1300 cm^−1^ spectral range, the IR band from 1710 cm^−1^ characteristic of C=O vibrations [32,33] in the IR spectrum of the HApCs target (see Figure 4a) is shifted to 1744 cm^−1^ in the spectrum of the film (Figure 4b). Further, the IR band characteristic of N-H vibrations [5] from 1558 cm^−1^ (Figure 4a b) is shifted to 1544 cm^−1^ (Figure 4b). The IR bands from ~ 1400 cm^−1^ due to [CO_3_]^2−^ groups [10] present in the target are strongly diminished in the film. The IR bands from 1395 and 1378 cm^−1^ are due to C-H vibrations in the chitosan structure.

The IR bands characteristic of P-O vibrational modes are broadened in the spectrum of the film in comparison with the spectrum of the target (Figure 4a,b). Therefore, peak fitting analysis is recommended to be performed for a proper identification of the molecular structure of HApCs layers.

The deconvolution of the IR molecular band positioned at around 1000 cm^−1^ in the HApCs coating revealed slight shifts from 1090, 1022 and 960 (in the deconvoluted IR spectrum of the target, Figure 4c) to 1095, 1022 and 940 cm^−1^ (in the deconvoluted IR spectrum of the film, Figure 4e). These bands are specific to P-O asymmetric and symmetric stretching vibrations in [PO_4_]^3−^groups of the apatite structure. It results that the processes in the plasma during the deposition influence not only the interaction between N-H/O-H bonds but also the P-O vibrations in the apatite structure. The deconvoluted IR bands from 1050 cm^−1^ (in the deconvoluted IR spectrum of the HApCs target, Figure 4c) and 1053 cm^−1^ (in the deconvoluted IR spectrum of the film, Figure 4e) can be assigned either to P-O stretching in [PO_4_]^3−^groups or to C-O stretching in chitosan. Usually, the saccharide structure of chitosan is manifested by a molecular band in the 1070–1028 cm^−1^ spectral domain [33]. Deconvoluted IR bands from 1004/995 cm^−1^ (in the IR spectra of the HApCs target/coating, Figure 4c,e) are ascertained to the C-H/C-O bond vibrations in the HApCs [32]. The IR band from 1107 cm^−1^ can indicate the P-O vibrations in the non-apatitic phosphate structure [34].

In the spectral range of 650–400 cm^−1^, the deconvoluted curves from 610, 584 and 557 cm^−1^ belong to P-O vibrations (O-P-O bending mode ν_4_) in [PO_4_]^3^.

The broadening of the molecular bands in the 700–500 cm^−1^ and 1200–900 cm^−1^ spectral ranges of the IR spectrum of HApCs layers in comparison with the IR spectrum of the HApCs target indicates the formation of interlinked bonds into the layer between phosphate groups of hydroxyapatite and C-H/C-O bonds specific to chitosan. Similar FTIR spectra were obtained for HApCs coatings generated at 2 × 10^−3^ mbarr and 1.2 × 10^−2^ mbarr Ar gas working pressures and 30 W rf power.

### 3.5. SEM Analysis of HAp–Chitosan Coatings

The features of the surface morphology of the HApCs coatings deposited on Si substrates in rf magnetron discharge were investigated by scanning electron microscopy.

As a source of generation of vaporized material, rf magnetron discharges are frequently used for synthesis of polymer nanoparticles in plasma [13]. The sputtering of a polymer target with Ar+ ions conduces the release into the plasma of volatile molecular fragments. Further, the three-step polymerization process that involves nucleation, coagulation and growth by accretion leads to formation of polymer nanoparticles with different size distributions [13,21]. The sizes and shapes of nanoparticles depend on the plasma chemistry and parameters such as: working gas type, flow and pressure or applied rf power [13,21].

**Figure 5:**
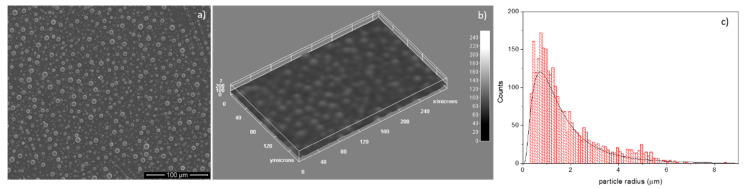
(**a**) Survey SEM image of HApCs coatings generated at 5 × 10^−3^ mbarr Ar gas working pressure; (**b**) 3D image of Figure 1 performed with ImageJ software; (**c**) histogram of particle sizes.

In this context, the adding of chitosan to hydroxyapatite (in the synthesis process), for its future use as a sputtering target in rf magnetron discharge, leads to different surface morphologies of HApCs coatings (see Figure 5a,b) in comparison with those of HAps [10,12]. Thus, on the surface of HApCs layers, grain-like structures with spherical shapes and various sizes are formed that can be assigned to the coagulation of macromolecules of HApCs (produced in the magnetron plasma), on the substrate. This mechanism can be explained by the huge temperature differences in the plasma (thousands of K) and at the substrate (523 K) during the deposition process. In Table 1, the plasma temperature values measured in different experimental conditions are shown. Figure 5c presents the grain size particle distribution extracted from the SEM image shown in Figure 5a.

**Figure 6:**
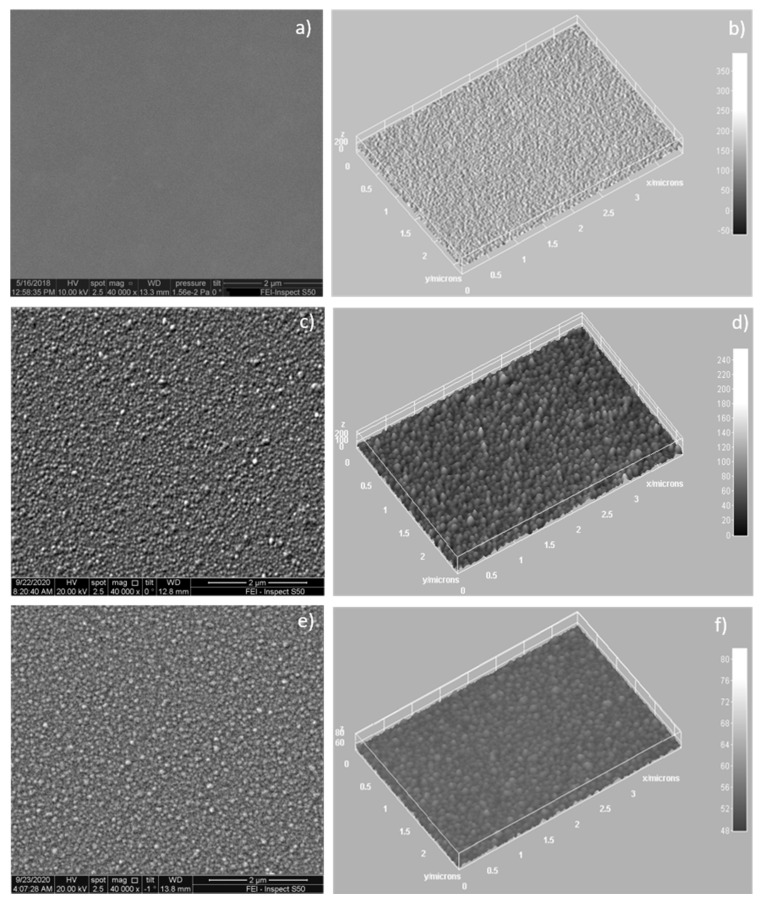
SEM images of HApCs coatings at: (**a**) p = 2 × 10^−3^ mbarr; (**c**) p = 5 × 10^−3^ mbarr; (**e**) p = 1.2 × 10^−2^ mbarr; 3D images of: (**b**) Figure 6a; (**d**) Figure 6c; (**f**) Figure 6e; performed with ImageJ software.

The SEM images acquired with 40,000× magnification reveal a compact granular structure of hundreds of nanometers of the HApCs coatings (see Figure 6). The 3D image analysis (see Figure 6b–f) indicates the evolution of the grain size as a function of the Ar gas pressure and consequently the energy and density of the particles that attain the coating surface during the deposition process. Thus, it was observed that the size of the spherical-shape grains increases and their heights decrease as the Ar gas pressure increases from 5 × 10^−3^ to 1.2 × 10^−2^ mbarr.

We suppose that this effect is possible as the density of the electron number in the plasma increases with the Ar gas working pressure (see Table 1), inferring that the density of charged particles which attain the surface of the coating increases too.

The nucleation stage of polymer vapors into nanometer-size particles is governed by the negative charges [13]. Their further coagulation in larger particles with tens of nanometers in diameter is dominated by a negative charge as the electrons have higher mobility than positive ions. Therefore, the density of the particles at the surface of the coating can influence the polymer nanoparticles formation and their shapes and sizes.

At 2 × 10^−3^ mbarr, the surface of HApCs layer is smooth because the energy of species moving from the plasma to the substrate is higher at lower pressures (see Table 1 and [10,19]).

### 3.6. EDS Analysis of HApCs Coatings

**Figure 7:**
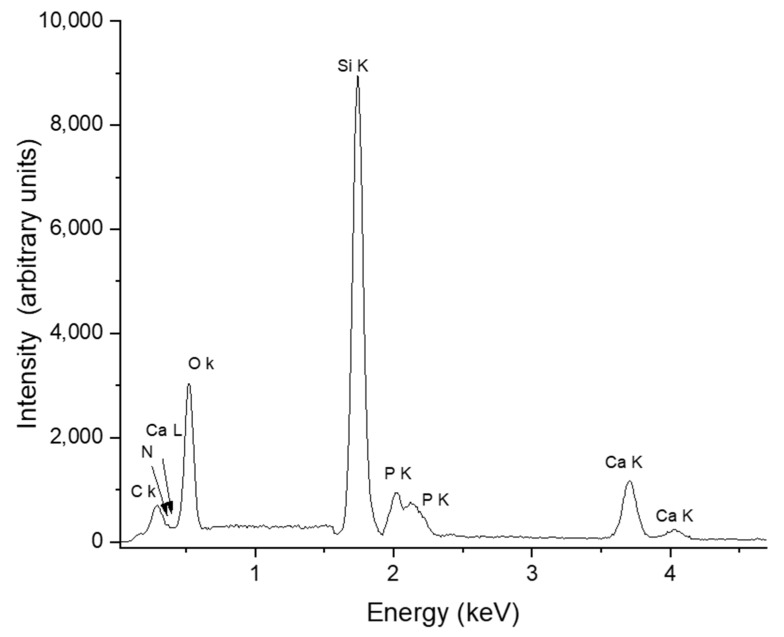
EDS spectrum of HApCs layers generated at 5 × 10^−3^ mbarr Ar gas working pressure.

The EDS spectrum of the HApCs layer is presented in Figure 7. The presence of Ca, O, P, C and N elements characteristic of both HAp and chitosan are revealed. The Ca/P atomic ratio was determined from EDS quantification measurements to be about 1.4.

**Figure 8:**
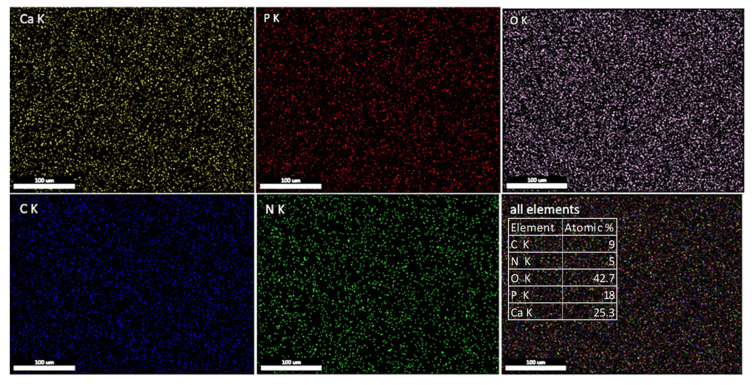
2D SEM-EDS elemental mapping of the HApCs coating.

The SEM-EDS elemental mapping of coatings revealed the distribution of the chemical elements characteristic of both hydroxyapatite and chitosan. Figure 8 shows the uniform distribution of Ca, P, O, C and N elements on the surface of the coatings. As the measurements were performed at 10 kV and in the EDS spectrum of the HApCs layer (see Figure 7), the Si coming from the substrate is highly visible, it results that the elemental data presented in Figure 8 are collected also from the sample volume. Therefore, it can be concluded that the Ca, P, O, C and N elements are homogeneously distributed in the sample volume.

The atomic percentages of all the elements in the analyzed area (see Figure 8) indicate a low content of nitrogen and carbon in the sample. These results can explain the reduced intensity of the N high-resolution XPS line (see Figure 3f).

## 4. Conclusions

The sputtering of a HApCs target in rf magnetron discharge at different Ar gas working pressures leads to the depositions of composite thin films of HApCs on silicon substrates. The analysis of the plasma by electrical measurements and residual gas mass spectrometry allowed the finding of electron temperatures of thousands of Kelvins, which explain the dissociation of molecules sputtered from the target and their future involvement in the growth mechanism of the films. Even if low rf powers and deposition rates were used, the difference between the temperature of the species in the plasma and the temperature at the sample holder favors the coagulation of HAp–chitosan macromolecules on the substrate and formation of layers whose surfaces present grain-like structures with sizes that vary from few microns to tens of nanometers. The nm sizes of the compact grain-like structures detected on the surface of the HApCs layers increase with the electron number density of the deposition plasma.

By X-ray photoelectron spectroscopy, we identified the chemical state of the films which correspond to a hydroxyapatite structure which is bound to the chitosan polymer.

The FTIR spectral analysis indicates molecular bands assigned to the vibrations of P-O bonds in the apatite structure as well as to the C-H and N-H bonds characteristic of chitosan.

## Figures and Tables

**Table 1:** Electron temperature and electron number density.

Ar gas Working Pressure (mbarr)	Ar Gas Flow (sln/min)	Rf Power	T_e_ (eV) HApCh (1 eV = 11,600 K)	n_e_ (m^−3^) HApCh
2 × 10^−3^	0.8	30 W	3.2	7.8 × 10^14^
5 × 10^−3^	2.4	30 W	2.5	9 × 10^14^
1.2 × 10^−2^	4.5	30 W	1.9	1 × 10^15^
2 × 10^−3^	0.8	50W	2.5	1.2 × 10^15^
5 × 10^−3^	2.4	50W	2.2	1.3 × 10^15^
1.2 × 10^−2^	4.5	50W	1.8	1.6 × 10^15^
2 × 10^−3^	0.8	70 W	2.9	1.5 × 10^15^
5 × 10^−3^	2.4	70 W	2.1	1.8 × 10^15^
1.2 × 10^−2^	4.5	70 W	1.7	1.9 × 10^15^
